# Cooperation of Rel family members in regulating A*β*_1-40_-mediated pro-inflammatory cytokine secretion by retinal pigment epithelial cells

**DOI:** 10.1038/cddis.2017.502

**Published:** 2017-10-12

**Authors:** Junran Sun, Peirong Huang, Jian Liang, Jie Li, Mengxi Shen, Xiangjun She, Yiji Feng, Xueting Luo, Te Liu, Xiaodong Sun

**Affiliations:** 1 Department of Ophthalmology, Shanghai General Hospital (Shanghai First People’s Hospital), School of Medicine, Shanghai Jiao Tong University, Shanghai, China; 2 Shanghai Engineering Center for Visual Science and Photomedicine, Shanghai 200080, China; 3 Shanghai Key Laboratory of Fundus Diseases, Shanghai, China; 4 Department of Ophthalmology, Shanghai Municipal Hospital of Traditional Chinese Medicine, Shanghai University of Traditional Chinese Medicine, Shanghai, China; 5 Department of Pathology, Yale University School of Medicine, New Haven, CT, USA; 6 Shanghai Geriatric Institute of Chinese Medicine, Longhua Hospital, Shanghai University of Traditional Chinese Medicine, Shanghai, China

## Abstract

Amyloid-beta (A*β*) is a hallmark component of age-related macular degeneration (AMD), which induces secretion of pro-inflammatory cytokines from retinal pigment epithelium (RPE). Previous studies have shown that p50/RelA (p65), a member of NF-*κ*B family, is an essential pro-inflammatory transcription factor responding to A*β*_1-40_ stimulation, but few focused on the other two Rel transcription factor members – RelB and c-Rel – and their role in A*β*_1-40_-mediated inflammation. It was reported that RelA, RelB and c-Rel are also implicated in various NF-*κ*B-mediated inflammatory diseases. Therefore, we infer that A*β*_1-40_-mediated inflammation targets not only the classical inflammation regulator, RelA, but also RelB and c-Rel. In this study, we demonstrate that intravitreally injected A*β*_1-40_ mice develop AMD-like pathologic changes, coupled with Rel protein (RelA, RelB and c-Rel) synthesis and nuclear translocation. To focus on the interaction mechanism of Rel proteins, we found that RelB and c-Rel formed a heterodimer with RelA in mice model. We also found that c-Rel silencing decreased the levels of A*β*_1-40_-dependent RelA expression, indicating that RelB and c-Rel may interact with RelA as coactivator and c-Rel is required to activate the expression of RelA. Moreover, Rel protein silencing decreased the expression of distinct pro-inflammatory cytokines. Together, we demonstrate that besides RelA, RelB and c-Rel can also be activated by A*β*_1-40_, all of which mediate pro-inflammatory cytokine transcription and RPE damage. Our findings imply that RPE-mediated inflammation under the stimulation of A*β*_1-40_ is multi-targeted and RelA, RelB and c-Rel proteins may be the new targets of anti-inflammatory agents.

Age-related macular degeneration (AMD) is one of the principal causes of visual impairment among aged population in developed countries.^1^ Therapies targeting vascular endothelial growth factor substantially improve the visual acuity of patients with exudative/wet AMD. Unfortunately, nonexudative/dry AMD, the other subtype of AMD that accounts for up to 90% of cases, has no established treatment.^2, 3, 4^ It is universally acknowledged that AMD has a multifaceted etiology. Excessive inflammation, including complement activation,^5^ inflammasome activation^6^ and accumulation of immune activators, is an important factor in the development of AMD. Despite advances in the understanding of AMD pathogenesis, the underlying mechanism of how inflammation facilitates the development of this disease is still under investigation.

The pathogenesis of early AMD is characterized by subretinal pigment epithelial deposits – drusen, which is composed of a range of proteins and lipids.^7^ Increasing evidences support that amyloid-beta (A*β*), one of the components of drusen, is correlated with the pro-inflammatory events in retinal pigment epithelial (RPE) cells.^8, 9^ Compared with other forms of A*β*, A*β*_1-40_ is the most prevalent type in drusen.^10^ Both subretinal and intravitreal injections (IVLs) of A*β* upregulate the expression of inflammatory mediators in RPE cells and develop AMD-like pathological changes.^9, 11^ The mechanism by which A*β* triggers inflammation in the retina is still not fully understood. It was reported that A*β*_1-40_ deposition triggered inflammation in the retina via activation of the NF-*κ*B pathway. Liu *et al.* demonstrated that NF-*κ*B/RelA activation was enhanced in RPE cells after the stimulation of A*β*_1-40_. The NF-*κ*B pathway is suppressed by vinpocetine, resulting in reduced expression levels of IL-1*β*, IL-18, NLRP3 and other cytokines.^12^ In addition, Lee *et al.* proved that A*β*_1-40_ induced activation of NF-*κ*B/RelA and TNF-*α* mRNA expression.^13^

Inducible transcription factors in the NF-*κ*B family exist as homodimers or heterodimers of five distinct proteins, including RelA (p65), RelB and c-Rel (Rel), p50 and p52, which contain a highly conserved N-terminal Rel homology domain. The p50 and p52 proteins are unable to activate transcription independently, as they lack transactivation domains. In contrast, RelA (p65), RelB and c-Rel, defined as the ‘Rel proteins’, have C-terminal transactivation domains.^14^ NF-*κ*B dimers normally stay inactive in the cytoplasm and transfer into the nucleus when they are activated. Despite the fact that p50 and p52 might have some potential effect on physiopathogenesis of inflammation, we explored a possible role of Rel proteins in A*β*_1-40_-mediated inflammation in the present study. As the most prototypical NF-*κ*B dimer, the p50/RelA heterodimer is associated with inflammation, oxidative stress and cellular homeostasis in numerous diseases, including AMD. In addition to RelA, the other two members of the Rel family, RelB and c-Rel, are primarily restricted to immune cells. RelB−/− or c-Rel−/− mice develop impaired cellular immunity,^15, 16^ suggesting their close connection with inflammation. However, the functions of RelB and c-Rel in an A*β*-induced AMD model have not yet been investigated. Therefore, in the current study, we proved that A*β*_1-40_ can activate RelA, RelB and c-Rel both *in vivo* and *in vitro*, all of which co-mediate inflammatory reactions. Our study has a implication that RPE-mediated inflammation under the stimulation of A*β*_1-40_ is multi-targeted, thus RelA, RelB and c-Rel proteins may be the new targets of anti-inflammatory agents.

## Results

### IVL of A*β*_1-40_ induces RPE damage and pro-inflammatory cytokine expression

**Figure 1:**
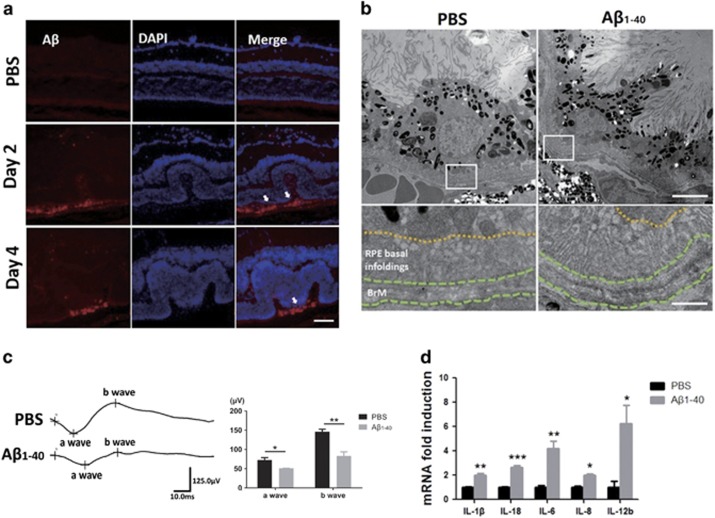
A*β*_1-40_ induces RPE impairment and upregulates pro-inflammatory cytokine expression *in vivo*. C57BL/6 mice were injected intravitreally with 5 *μ*l of 2.8 *μ*g/*μ*l A*β*_1-40_ (oligomeric form) or vehicle (PBS). (**a**) Immunoreactivity for A*β*_1-40_ was detected in the OS and RPE cells (arrows) at days 1 and 4 in the A*β*-injected sections. Scale bar: 50 *μ*m. (**b**) TEM of the RPE and Bruch’s membrane regions in mice. Obvious thickening of the BrM and RPE basal in-foldings with ultrastructural alterations (Scale bar: 5 *μ*m) was observed compared with that in the PBS-injected control mice (Scale bar: 1 *μ*m). (**c**) Demonstration of waveforms of the maximal ERG response and amplitude evaluations of the scotopic ERG responses were recorded. (**d**) IL-1*β*, IL-18, IL-6, IL-8 and IL-12b mRNA expression levels in RPE–choroid in response to A*β*_1-40_ at day 4. BrM, Bruch’s membrane; CC, choriocapillaris; OS, outer segment. Histograms represent the mean and S.E.M. *N*=8, NS=nonsignificant *P*-value, *P<0.05, **P<0.01, ***P<0.001 via Student’s *t*-tests

The penetration of intravitreally injected A*β*_1-40_ has been proved through rat retinas^9, 17^ but not through the retinas of C57BL/6 mice. To verify the deposition of A*β*_1-40_ on RPE cells, we performed immunofluorescence on retinal sections. In contrast to vehicle-injected eyes, increased A*β* signaling was observed at days 2 and 4 after IVL, and it was distributed predominantly in the photoreceptor outer segment and RPE cells (Figure 1a). This finding suggests that A*β*_1-40_ can spread widely through the mice retina and reach the RPE cells. To assess whether the A*β*_1-40_ deposition induces RPE cell senescence, transmission electron microscopy (TEM) was used to examine the RPE structures. The RPE cells in the phosphate-buffered saline (PBS)-injected mice appeared normal in morphology, whereas the RPE in the A*β*_1-40_ mice exhibited damage structures (Figure 1b). Specifically, both the BrM and RPE basal in-foldings were thicker, which were similar to the ultrastructural alterations in APOE4-HFC-induced AMD models, in which the expression of A*β*_1-40/42_ elevated.^18^ To test whether or not visual function was impaired by A*β* peptide, electroretinogram (ERG) was performed at day 4 since the injection. Significant reductions in the scotopic a- and b-wave ERG amplitudes in the A*β*_1-40_-injected mice were detected compared with those in control group, with 29% and 43% average decrease in the a and b waves, respectively (Figure 1c). The reduction in a-wave amplitudes suggests the impairment in photoreceptor and RPE. Next, we isolated RPE–choroid complexes and measured cytokine mRNA expression by qPCR after the injection of A*β*_1-40_. The expression level of five cytokines significantly increased under the A*β*_1-40_ stimulation on day 4: IL-1*β* (2.0±0.1-fold); IL-6 (4.2±0.6-fold); IL-8 (2.0±0.1-fold); IL-18 (2.7±0.1-fold); and IL-12b (6.2±1.5-fold; Figure 1d).

Altogether, these data suggest that the IVL of A*β*_1-40_ resulted in inflammation, which led to AMD-like pathology and induced an impaired visual function in mice.

### Rel protein levels increased in the A*β*_1-40_-mediated AMD model

**Figure 2:**
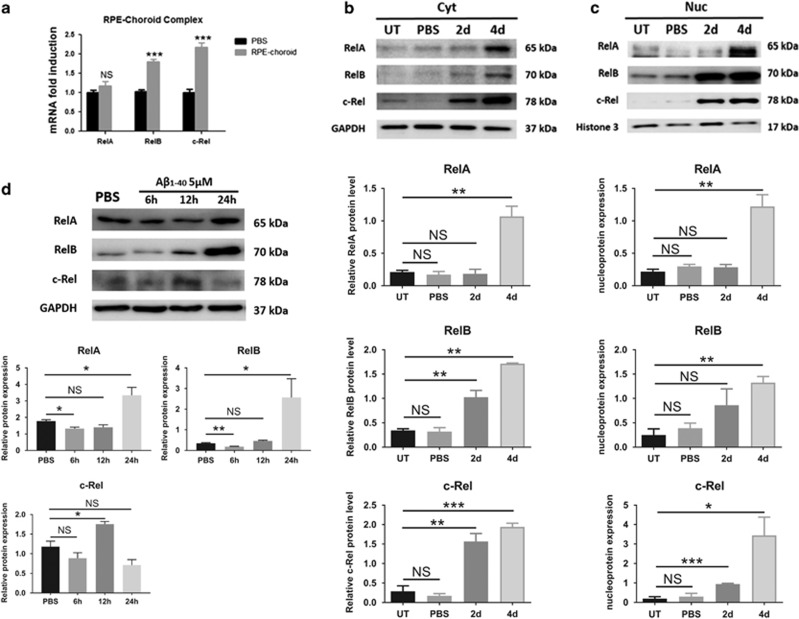
A time-dependent Rel protein expression pattern was induced by A*β*_1-40_ both *in vivo* and *in vitro*. (**a**) qRT-PCR assays of Rel gene expression in RPE–choroid complexes. Western blots showing time-dependent expression of Rel proteins in cytoplasmic (**b**) and nuclear extracts (**c**) of WT RPE–choroid complexes treated with 5 *μ*l of 2.8 *μ*g/*μ*l A*β*_1-40_ or PBS (0, 2 and 4 days). Histone H3 was used as a quality control for the nuclear extracts. (**d**) Rel protein expression in mouse RPE cells in response to 5 *μ*M A*β*_1-40_ at 6, 12 and 24 h after treatment. UT, untreated; WT, wild type. Histograms represent the mean and S.E.M. *N*=3, NS=nonsignificant *P*-value, *P<0.05, **P<0.01, ***P<0.001 via Student’s *t*-tests

It is known that RelA is an enhancer of A*β*-induced cytokine transcription in RPE cells; however, RelB and c-Rel are also the key regulators of numerous pro-inflammatory cytokines.^19, 20^ To investigate whether A*β*_1-40_ affects Rel protein expression *in vivo*, Rel mRNA expression in the RPE–choroid complex was evaluated by quantitative real-time RT-PCR. Although there were no significant differences in RelA mRNA levels, the RelB mRNA levels were upregulated (1.81±0.06-fold), and the c-Rel mRNA levels increased (2.30±0.12-fold) at day 2 (Figure 2a). Next, Rel protein expression was assessed in RPE–choroid complex cytoplasm exposed to A*β*_1-40_ at different time points. Compared with the controls, A*β*_1-40_ treatment resulted in significant increases in the RelB and c-Rel cytoplasm expression levels at day 2, followed by an elevation in RelA levels at day 4 (Figure 2b). In addition, increased RelB and c-Rel nucleoprotein expression levels were observed at day 2, followed by a constant high expression level until day 4, whereas RelA was upregulated only at day 4. It suggests a different time pattern of Rel protein activation, which is consistent to the Rel protein expression in cytoplasm described above (Figure 2c). We further investigated the potential role of A*β*_1-40_ in primary mouse RPE cells. The primary mouse RPE cells were measured at 6, 12 and 24 h of culture in the presence of 5 *μ*M A*β*_1-40_. RelB and c-Rel were significantly upregulated in RPE cells at 12 h. After 24 h, the expression level of c-Rel decreased; however, the RelB level still remained higher than the controls. The RelA level was the same between the treatments and the time before 12 h, but they started to elevate at 24 h (Figure 2d). Our data show that the cytoplasmic and nuclear Rel protein levels were elevated following A*β*_1-40_ stimulation in a time-dependent pattern.

### A*β*_1-40_ triggers Rel nuclear translocation and activation

To figure out the effect of the Rel proteins on the activation of pro-inflammatory cytokine transcription, we detected alterations in Rel protein distribution in response to A*β*_1-40_ stimulation. Initially, we confirmed the nuclear translocation of the Rel proteins at the different time points as described above (Figures 2b and c).

**Figure 3:**
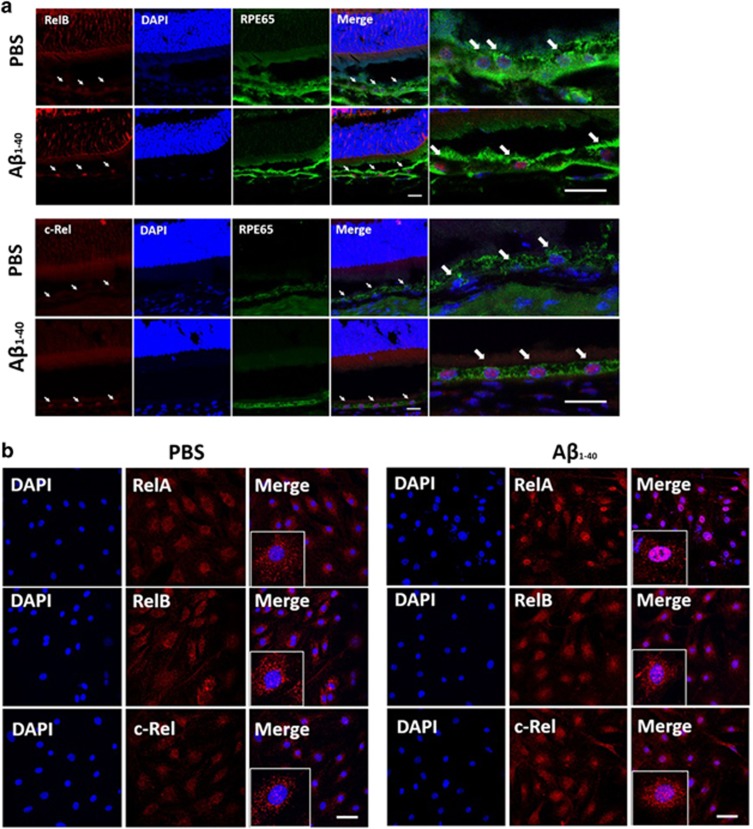
Rel protein translocation in RPE–choroid complexes and in primary mouse RPE cells. (**a**) Retinal sections co-immunostained with RPE65 (green) and RelB/c-Rel(red) at day 4. RPE nuclei were marked by white arrows (Scale bar: 20 *μ*m). (**b**) Confocal microscopic analysis showing the distribution of the RelA/RelB/c-Rel signal (red) at 24 h after 5 *μ*M A*β*_1-40_ or PBS treatment. Nuclei were immunostained with DAPI (blue) (Scale bar: 50 *μ*m)

Next, we used confocal microscopic analysis to detect Rel protein nuclear translocation. RelB and c-Rel showed strong fluorescence signal in the nuclei of RPE cells, which were marked by RPE65 at day 4, whereas no nuclear signaling was observed in the PBS group (Figure 3a). For further confirmation, primary mouse RPE cells were incubated with A*β*_1-40_ for 24 h. Nuclear Rel protein expression was hardly observed in the PBS group; however, the nuclear fluorescence signal significantly increased in the A*β*_1-40_ group (Figure 3b). These results indicate that Rel protein translocation into the nucleus can be triggered by A*β*_1-40_ in a periodic manner.

### A*β*_1-40_ promotes c-Rel-dependent expression of RelA and the formation of RelA–RelB/c-Rel complexes

**Figure 4:**
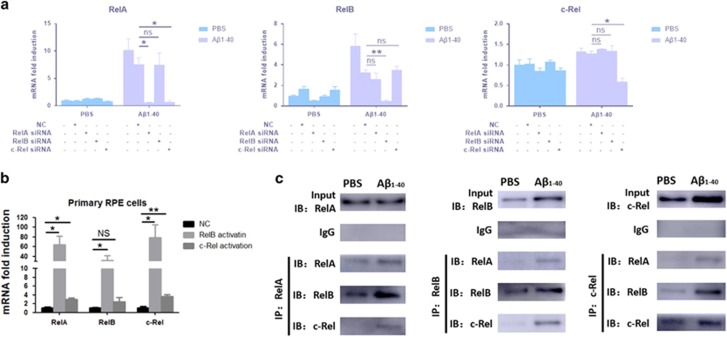
The interaction between Rel proteins in RPE–choroid complex and in RPE cells. (**a**) RelA, RelB and c-Rel silencing induced changes in the RelA, RelB and c-Rel expression levels in ARPE-19 cells, as determined by qRT-PCR analysis. (**b**) qRT-PCR assays of Rel mRNA expression induced by RelA, RelB and c-Rel overexpression in primary mouse RPE cells. (**c**) Co-immunoprecipitation of c-Rel, RelB and RelA at 4 days post injection of A*β*_1-40_ or PBS. Histograms represent the mean and S.E.M. *N*=3, NS=nonsignificant *P*-value, *P<0.05, **P<0.01 via Student’s *t*-tests

To assess whether RelB and c-Rel were associated with the expression of RelA, siRNAs targeting RelB and c-Rel were transfected into ARPE-19 cells to silence their expression. After 48 h of siRNA treatment, the ARPE-19 cells were stimulated with A*β*_1-40_. As shown in Figure 4a, c-Rel silencing decreased the A*β*_1-40_-dependent RelA expression level, which was similar to those achieved by RelA silencing alone. In contrast, RelA silencing had no effect on either RelB or c-Rel expression, regardless of exposure to A*β*_1-40_ (Figure 4a). In addition, RelB or c-Rel overexpression by transfection with their respective lentivirus activation particles led to a increase in the other two Rel protein members in primary mouse RPE cells (Figure 4b). These results suggest that there is a crosstalk and a common pathway among the Rel proteins and c-Rel in specific positively regulates the expression of RelA after A*β*_1-40_ stimulation in RPE cells.

Furthermore, RelA may be an activator as part of the RelA–RelB/c-Rel dimer. Therefore, RelA in the RPE–choroid complex was co-immunoprecipitated with RelB and c-Rel at day 4 after injection. The data strongly indicate that RelB and c-Rel formed heterodimers with RelA after IVL of A*β*_1-40_ (Figure 4c). In summary, these results suggest that RelA activity might be affected by the preferential nuclear translocation of the dimers RelA–RelB and RelA–c-Rel, and the expression of c-Rel in A*β*_1-40_-stimulated RPE cells.

### Rel proteins are the intracellular messengers of A*β*_1-40_-mediated inflammation

**Figure 5:**
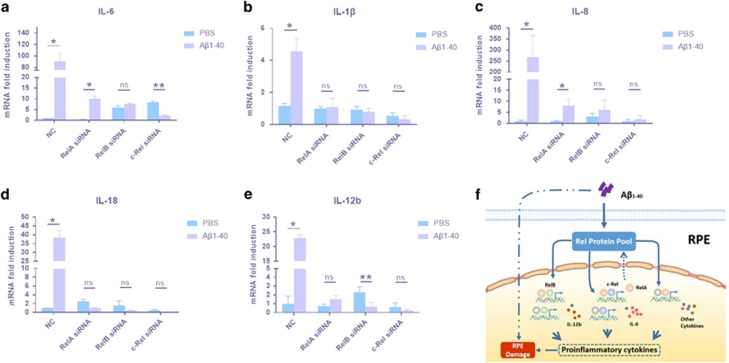
Effects of Rel protein silencing on pro-inflammatory cytokine levels in RPE cells. (**a**–**f**) ARPE-19 cells were transfected with only Lipofectamine 6000 reagent, non-siRNA, RelA siRNA, RelB siRNA or c-Rel siRNA for 24 h. IL-6 (**a**), IL-1*β* (**b**), IL-8 (**c**), IL-18 (**d**) and IL-12b (**e**) mRNA levels were evaluated by qRT-PCR. The mRNA levels were standardized to housekeeping gene expression. (**f**) Schematization of the model obtained by the integration of the data obtained in this report and the context. Histograms represent the mean and S.E.M. *N*=3, NS=nonsignificant *P*-value, *P<0.05, **P<0.01 Student’s *t*-tests

To confirm whether RelB/c-Rel are key mediators of pro-inflammatory cytokines, as RelA is, we silenced their expression in ARPE-19 cells. Consistent with the above *in vivo* results, IL-1*β*, IL-8, IL-18, IL-6 and IL-12 mRNA levels were upregulated in ARPE-19 cells 24 h after exposure to A*β*_1-40_. The statistical analysis showed the significant differences in Rel protein expression levels following siRNA treatment compared with those in control siRNA. The IL-1*β*, IL-18 and IL-12b in the A*β*_1-40_ group decreased to a similar level in RelA, RelB and c-Rel knocked-down RPE cells in contrast to control siRNA, whereas IL-6 and IL-8 mRNA were downregulated in the RelB- and c-Rel-silenced groups to a greater degree than RelA, despite partial inhibition in the RelA-silenced group (Figures 5a–e). Moreover, A*β*_1-40_-induced IL-6 expression significantly decreased only in the c-Rel-silenced RPE cells (Figure 5a), and A*β*_1-40_-induced IL-12b expression significantly decreased only in the RelB-silenced RPE cells (Figure 5e). These results indicate that although RelA is central to these processes in A*β*-mediated RPE inflammation, RelB and c-Rel silencing also decreases cytokine expression to a greater extent, suggesting a different RelB/c-Rel-dependent mode of regulation. The c-Rel-mediated regulation of IL-1*β*, IL-18 and IL-12b expression may depend on RelA according to the results above, which needs further investigation. The schematic diagram of Rel proteins in pathogenesis of A*β*_1-40_-mediated inflammation is illustrated in Figure 5f.

## Discussion

In this study we demonstrate that A*β*_1-40_ activates the pro-inflammatory transcription factor Rel proteins *in vivo* and in RPE cells. The present data also show that not only RelA but also RelB and c-Rel are activated, and RelB and c-Rel form heterodimers with RelA in a A*β*_1-40_-related AMD model. Our study established for the first time that the effects of A*β*_1-40_ in RPE-mediated inflammation are multi-targeted.

First, we proved that intravitreally injected A*β*_1-40_ reaches RPE cells, which results in an impairment of the retina. Our prior study showed that RPE cells from A*β*_1-40_-injected mice displayed hyper- and hypopigmentation.^21^ In the current study, the structure and function of the retina were evaluated by TEM and ERG, respectively. The ultrastructural alterations and decreased ERG responses observed in our study were similar to those reported in other AMD animal models.^22, 23^ However, these studies investigated only the effects of certain risk factors in AMD development, such as pathologic changes. As there are no generally established AMD animal models we focused on the influence of A*β*_1-40_ on RPE cells in this study. The model we used may partially explain the association between A*β*_1-40_ and retinal degeneration in AMD.

Our previous study showed that RelA and the I*κ*B kinase inhibitor of *κ*B kinase epsilon, which are essential in regulating NF-*κ*B-mediated inflammatory responses, were implicated in informatics analyses of chip results in an A*β*_1-40_-injected model.^21^ Subsequent to our prior results, the current study provided evidence that A*β* activates transcription factor Rel proteins in a time-dependent pattern *in vivo* and in RPE cells. Given that RelA has been well studied in A*β*_1-40_-mediated inflammation, we focused on the biological functions of RelB and c-Rel in this study. We demonstrated that A*β*_1-40_ induces increased translocation of RelB and c-Rel into the nucleus before RelA, whose expression may be regulated by c-Rel (Figures 2b and c). The time-dependent pattern of Rel protein expression has also been reported in other studies,^24, 25^ which leads to the hypothesis that RelB and c-Rel, along with RelA, might have important roles in different time periods;^26^ however, this possibility requires further investigation. In addition, the association of RelA with RelB and c-Rel was markedly increased in response to A*β*_1-40_ stimulation in RPE cells (Figure 5c). Previous reports have suggested that distinct combinations of Rel proteins generate specific NF-*κ*B-responsive gene profiles by affecting transcriptional activation, although the Rel dimer composition depends on the cell type and cellular stimuli. Jacque and colleagues described inhibition of RelA DNA binding by RelA–RelB complex formation after lipopolysaccharide (LPS) stimulation in lymphoid cells.^27^ RelA–c-Rel dimers initiated transcriptional amplification of c-Rel and the formation of c-Rel–c-Rel dimers, which exert protective effects on RPE cells.^28^ Therefore, it is likely that RelB and c-Rel may affect cytokine expression through the following mechanisms: (a) modulation of RelA activity by direct complex formation; (b) modulation of RelA expression; and (c) directly binding to cytokine promotors without RelA in A*β*_1-40_-stimulated RPE cells.

We have shown that Rel proteins enhance pro-inflammatory cytokine secretion in RPE cells. There is a strong link between these cytokines and AMD in patients.^29, 30^ Maturation of IL-1*β* and its analog, IL-18, was promoted by NLRP3 inflammasome activation, a pathway essential in both dry and wet AMD.^31^ IL-1*β* and IL-18 have cytotoxic effects in inducing RPE degeneration. In addition, IL-1*β* promotes neovascularization,^32^ whereas contrasting findings have been reported that IL-18 inhibits angiogenesis in choroidal neovascularization.^33^ Thus, whether exposure to IL-1*β* and IL-18 results in AMD-like pathology remains an important question. IL-6 and IL-8 are prominent cytokines that mediate chronic inflammatory responses shared across a variety of age-related pathologies and are implicated in AMD progression following LPS stimulation.^34, 35^ IL-6 also had a pro-angiogenic effect in an AMD model, similar to IL-1*β* and IL-18.^36^ IL-12b upregulation may also be a risk factor in AMD.^37^ These cytokines can enhance the synthesis of other cytokines, suggesting that the A*β*_1-40_ toxicity mediated by these cytokines through an autocrine feedback loop may impair RPE cells and stimulate angiogenesis, contributing to AMD pathogenesis. Among them, IL-1*β*, IL-18 and IL-12b secretion levels were impaired in RelA- or c-Rel/RelB-silenced RPE cells, whereas the IL-6 and IL-8 mRNA levels were diminished only in the c-Rel- or RelB-deleted RPE cells, but not in the RelA-knockdown RPE cells. These two different regulation patterns by the Rel proteins on these two cytokine expression profiles may reveal their different functions in AMD, although their underlying mechanisms remain unknown. However, conflicting results have also been reported that RelB recruitment to target genes is associated with transcriptional downregulation of IL-12b, while our data indicated that the IL-12b mRNA level was significantly decreased in the RelB-silenced group (Figure 5e). These controversial results are perhaps due to the different roles of RelB in distinct cells or stimuli.^38^ DNA binding of Rel proteins to cytokine promoters requires further exploration. We infer that therapy targeting RelB/c-Rel may be a more effective strategy to reduce A*β*_1-40_-mediated RPE damage.

In summary, our data confirm the following hypotheses: (1) activation of RelA, RelB and c-Rel occurs in A*β*_1-40_-stimulated RPE cells; (2) activated Rel proteins positively regulate inflammation; and (3) inflammation is alleviated in RPE cells as long as one of the Rel proteins is knocked out. Three Rel proteins may now be considered important regulators of A*β*_1-40_-induced RPE degeneration. These findings indicate the multi-targeted effects of A*β*_1-40_, which shed light on endogenous signaling in A*β*-induced AMD. Hopefully, it can provide novel therapeutic approaches for RPE protection in AMD.

## Materials and methods

### Amyloid oligomerization

A*β*_1-40_ oligomeric peptide (GL Biochemistry, Shanghai, China) was prepared as previously described.^39^ Briefly, synthetic lyophilized A*β*_1-40_ peptides were dissolved in deionized distilled water at a concentration of 28 *μ*g/*μ*l. A*β* solutions were diluted to reach a final concentration of 2.8 *μ*g/*μ*l by immediate addition of PBS and incubated for 7 days at 37 °C. The samples were stored at −80 °C for use. Electron microscopy was used to verify the aggregated states of A*β*_1-40_.

### Animal model and treatment

Two-month-old male C57BL/6 mice were supplied by the Laboratory Animal Center at the Shanghai First People’s Hospital. The animals were reared and housed in sterilized enclosures with a 12 h light cycle.

The mice were anesthetized with 1.5% sodium pentobarbital (100 *μ*l/20 g intraperitoneally (i.p.)) and were administered a single unilateral IVL of A*β*_1-40_ peptides (14 *μ*g/5 *μ*l) in PBS using a glass micropipette to deliver oligomeric A*β*_1-40_ peptides under a dissecting microscope (SM2000J; Olympus, Tokyo, Japan). The A*β*_1-40_ peptide concentration was reported in a previous study.^21^ Briefly, age-matched controls received 5 *μ*l of PBS in the same manner (*n*=8). The mice eyes were enucleated under i.p. anesthesia after 2 and 4 days since the injection. All animal experiments were approved by the Ethics Committee of Jiao Tong University, Shanghai, China, and were conducted in compliance with the Association for Research in Vision and Ophthalmology Statement for the Use of Animals in Ophthalmic and Vision Research.

### Primary mouse RPE cell isolation and culture

To isolate RPE cells from 3-week-old wild-type C57BL/6 mice for primary culture, the anterior portion of the eye and retina were removed gently with forceps, and then 0.25% trypsin (Gibco, Carlsbad, CA, USA) was added to the eyecups for 20 min at 37 °C in a 5% CO_2_ atmosphere. After trypsin treatment, the RPE sheets were peeled off under a dissecting microscope. The collected single-cell suspension was transferred to complete Dulbecco’s modified Eagle’s medium /Ham’s F-12 medium (Gibco) supplemented with 10% fetal bovine serum, 1% non-essential amino acids and 1% HEPES (Gibco). The cells were passed every 3 days by 0.25% trypsin (Gibco).

### Electroretinography

Scotopic ERG was performed using RETIport System (Roland Consult, Brandenburg, Germany) with a Super Color Ganzfeld (Q450 SC) stimulator. The animals were dark-adapted overnight, and then were anesthetized with an i.p. of 1% sodium pentobarbital under red illumination. Pupils were dilated with atropine (0.5%). Contact lens electrodes were placed on the cornea following topical anesthesia of the cornea. The ground electrode was placed midway up the tail, and then the scotopic ERG was recorded. All manipulations were done under scotopic conditions. The amplitude of the a wave/b wave, defined as the scope from the baseline to the respective trough, was measured and analyzed using a built-in software.

### Real-time quantitative PCR

Total RNA extraction and quantification were performed according to the RNAsimple Total Kit protocol (Tiangen Biotech, Beijing, China). NanoDrop 2000c spectrophotometer (Thermo Fisher Scientific, Wilmington, DE, USA) was used to quantify the RNA samples. Following RNA extraction, RT Master Mix (Takara Bio Inc., Dalian, China) was used to generate cDNA with according to the manufacturer’s protocol.

The primers were as follows: glyceraldehyde-3-phosphate dehydrogenase (GAPDH) was used to normalize all samples. The specific sense and antisense primer sequences used are available Table 1. The RT-PCRs by a SYBR green-based PCR method were performed using a real-time PCR detection system (Eppendorf, Hamburg, Germany) with a program of 40 cycles of amplification (95 °C for 5 s, 60 °C for 30 s and 72 °C for 42 s). The relative expression level of each mRNA was calculated using the 2−ΔΔCt method.

### Western blot analysis

RPE–choroid tissues or RPE cells were collected and lysed with radio-immunoprecipitation assay buffer containing proteinase inhibitors. Nuclear and cytoplasmic protein were extracted using Nuclear and Cytoplasmic Protein Extraction Kit (Beyotime, Shanghai, China). Aliquots of each sample were subjected to 10% SDS-PAGE gels and electroblotted to polyvinylidene difluoride membranes (Merck Millipore, Billerica, MA, USA). The membranes were blocked by blocking buffer (Tris-buffered saline Tween-20 (TBST), containing 5% nonfat dry milk) for 1 h at room temperature and incubated with primary antibodies against RelA (1:1000, Cell Signaling Technology, Beverly, MA, USA), RelB (1:2000, Abcam, Cambridge, MA, USA), c-Rel (1:2000, Abcam), GAPDH (1:1000, Cell Signaling Technology) or Histone H3 (1:1000, Cell Signaling Technology) overnight. The membranes were then washed with TBST three times, and then probed with horseradish peroxidase-conjugated secondary antibodies (1:2000, Proteintech, Chicago, IL, USA) for 1 h at room temperature. The membranes were washed with TBST and then were exposed to a molecular imaging system (Amersham Imager 600, GE Healthcare, Buckinghamshire, UK).

### Immunocytochemistry and TEM

Immunocytochemistry assays were performed on retinal sections or in 24-well slide chambers. Briefly, after fixation, the samples were blocked with 0.3% Triton X-100 and 5% goat serum albumin (Beyotime) in PBS for 1 h at room temperature. Tissue sections were immunostained with primary antibodies against RelA (1:300, Cell Signaling Technology), RelB (1:300, Abcam), c-Rel (1:300, Abcam) and RPE65 (1:300, Novus Biologicals, Littleton, CO, USA) overnight at 4 °C. The samples were washed with PBS three times and then were stained for 45 min at 37 °C with Alexa Fluor 594- and 488-conjugated secondary antibodies (1:1 000, Proteintech). The nuclei were marked by 4′,6-diamidino-2-phenylindole. RPE cells were visualized using a fluorescence microscope (Olympus) and a Leica TCS SP8 confocal laser scanning microscope (Leica TCS NT, Wetzlar, Germany).

For ultrastructural analysis, the samples were send to Shanghai FuDan University School of Medicine after fixation in 2.5% glutaraldehyde (dissolved in PBS) at 4 °C. The ultrathin sections were observed under an electron microscope (Tecnai G2 spirit twin, FEI, Eindhoven, The Netherlands).

### Lentiviral activation particles

Primary RPE cells were transduced with lentiviral-mediated RelB-specific CRISPR/dCas9 activation plasmid (Santa Cruz Biotechnology, Santa Cruz, CA, USA). Activated clones were selected via puromycin dihydrochloride (Santa Cruz Biotechnology) administration at 4 days after the transduction. RNA was isolated for activation analysis by RT-PCR 2 days later.

### RNA interference

ARPE-19 cells were transfected with double-stranded siRNA or negative control siRNA (non-siRNA) using Lipo6000 Transfection Reagent (Beyotime). Target sequences were as follows: 5′-GCCCUAUCCCUUUACGUCA-3′ for p65, 5′-GAGGACAUAUCAGUGGUGUUCAGCA-3′ for RelB and 5′-AAAUGUGAAGGGCGAUCAGCA-3′ for c-Rel. Double-stranded siRNAs were synthesized by Shanghai GenePharma (Shanghai, China).

### Co-immunoprecipitation and immunoblotting

Co-immunoprecipitation studies were performed on RPE–choroid complexes according to the Pierce Co-Immunoprecipitation Kit (Rockford, IL, USA) protocol. The following antibodies, rabbit anti-RelA (Cell Signaling Technology), rabbit anti-RelB/c-Rel (Abcam) or rabbit IgG (Santa Cruz Biotechnology), were used for immunoprecipitation. The immunoprecipitation products were analyzed via western blot. The following antibodies, mouse anti-RelA (Cell Signaling Technology) or mouse anti-Relb/c-Rel (Santa Cruz Biotechnology), were used for western blot analysis.

### Statistical analysis

Each experiment was repeated in triplicate. The data were expressed as the mean±S.E.M., if applicable. Two-tailed unpaired Student’s *t*-tests (GraphPad Prism, San Diego, CA, USA) were used to calculate the *P*-value of the results. A *P*-value <0.05 was considered to be statistically significant.

## Figures and Tables

**Table 1:** Quantitative RT-PCR primers

**Gene**	**Forward primer**	**Reverse primer**
Mouse GAPDH	5′-CGGAGTCAACGGATTTGGTCGTAT-3′	5′-AGCCTTCTCCATGGTGGTGAAGAC-3′
Mouse RelA	5′-GCCCAGACCGCAGTATCC-3′	5′-GTCCCGCACTGTCACCTG-3′
Mouse RelB	5′-CTGGCTCCCTGAAGAACC-3′	5′-CGCTCTCCTTGTTGATTC-3′
Mouse c-Rel	5′-CTCTGCCTCCCATTGTTTCTA-3′	5′-GGCTTCCCAGTCATTCAACAC-3′
Mouse IL-18	5′-CAGGCCTGACATCTTCTGCAA-3′	5′-CTGACATGGCAGCCATTGT-3′
Mouse IL-1*β*	5′-AGTTGACGGACCCCAAAAGAT-3′	5′-GTTGATGTGCTGCTGCGAGA-3′
Mouse IL-8	5′-CAAGGCTGGTCCATGCTCC-3′	5′-TGCTATCACTTCCTTTCTGTTGC-3′
Mouse IL-6	5′-CTTCCATCCAGTTGCCTTCTTG-3′	5′-AATTAAGCCTCCGACTTGTGAAG-3′
Mouse IL-12b	5′-ATGGAGTCATAGGCTCTGGAAA-3′	5′-CCGGAGTAATTTGGTGCTTCAC-3′
Human GAPDH	5′-TGTAGACCATGTAGTTGAGGTCA-3′	5′-AGGTCGGTGTGAACGGATTTG-3′
Human RelA	5′-CAGGCTCCTGTGCGTGTCTC-3′	5′-CTGGCTGATCTGCCCAGAAG-3′
Human RelB	5′-AGATTGAGGCTGCCATTGAG-3′	5′-CGCAGCTCTGATGTGTTTGT-3′
Human c-Rel	5′-CCATGTTCATCAGGGAGAAA-3′	5′-GCAGGAATCAATCCATTCAA-3′
Human IL-18	5′-AGTCAGCAAGGAATTGTCTCC-3′	5′-GAAGCGATCTGGAAGGTCTG-3′
Human IL-1*β*	5′-TTACAGTGGCAATGAGGATGAC-3′	5′-TGTAGTGGTGGTCGGAGATTC-3′
Human IL-8	5′-ATGACTTCCAAGCTGGCCGT-3′	5′-TCCTTGGCAAAACTGCACCT-3′
Human IL-6	5′-GATGGCTGAAAAAGATGGATGC-3′	5′-TGGTTGGGTCAGGGGTGGTT-3′
Human IL-12b	5′-ACCTGACCCACCCAAGAACT-3′	5′-GGACCT GAACGCAGAATGTC-3′
